# Improving blood transfusion safety in a low-resource setting: an audit of 1,163 transfusion requests

**DOI:** 10.1186/cc14417

**Published:** 2015-03-16

**Authors:** S Kudsk-Iversen, R Colhoun, D Chama, J Mulenga, MD Bould, J Kinnear, D Snell

**Affiliations:** 1 Zambia Anaesthesia Development Project, Lusaka, Zambia; 2 Zambia National Blood Transfusion Service, Lusaka, Zambia

## Introduction

Sub-Saharan Africa suffers from more acute life-threatening indications for blood transfusion compared with high-income countries [1]. The commonest 'systems failure' contributing to perioperative death in low-resource settings is the timely availability of correctly cross-matched blood products [2]. Often this is not the result of an absolute shortage of blood products, but failure in the chain of supply and distribution. We audited an early step in this chain, the quality of blood requests, at the University Teaching Hospital (UTH) in Lusaka, Zambia. UTH does not have a formal blood request form, and only the cancer diseases hospital (CDH) has a blood request form developed by the blood bank.

## Methods

We performed a 1-day retrospective review in June of blood request forms submitted to the cross-match laboratory, followed by a 14-day prospective review in September 2014. Group and save requests were excluded. Each form was audited against the American Association of Blood Banks (AABB) minimum standards for content of a blood request form. Analysis was performed with Fisher's exact test for nominal data and *t *test for continuous data.

## Results

A total of 1,163 blood requests were reviewed, 51 from CDH and 1,112 from other wards. Eighteen forms from CDH (35%) and 22 from other wards (2%) met all minimum AABB standards (*P *< 0.0001). The mean number of standards met on the requests from CDH and the rest were 11.25 (SD 0.93) and 8.87 (SD 1.75) respectively (*P *< 0.0001). Considering all blood requests, the standards met in order from least to most were: signature of requesting doctor (36%), urgency of request (43%), hospital number (59%), indication for transfusion (62%), type of product requested (72%), requesting doctor's name (78%), age or date of birth of patient (84%), gender of patient (89%), quantity of products requested (90%), date form was completed (90%), patient's ward (95%), and patient's full name (100%).

## Conclusion

The audit revealed an important system failure impacting on efficacy and safety of transfusion practice at UTH. Full patient identifiers, as well as vital information such as the indication and urgency, were rarely filled in, which are crucial for the blood bank to prioritise the release of blood products. The audit shows that practice may be significantly improved by a cheap intervention such as a standardised blood request form meeting international standards.

## Acknowledgements

UK aid, THET.

## References

[B1] Lund Transfus Apher Sci 2013 49 416 21 10.1016/j.transci.2013.06.014 23871466

[B2] Ohanaka Turk J Med Sci 2007 37 219 22

